# Epidemiological Characteristics of Lower Extremity Cellulitis after a Typhoon Flood

**DOI:** 10.1371/journal.pone.0065655

**Published:** 2013-06-13

**Authors:** Pei-Chen Lin, Hung-Jung Lin, How-Ran Guo, Kuo-Tai Chen

**Affiliations:** 1 Emergency Department, Chi-Mei Medical Center, Liouying, Tainan, Taiwan; 2 Emergency Department, Chi-Mei Medical Center, Tainan, Taiwan; 3 Department of Biotechnology, Southern Tainan University of Technology, Tainan, Taiwan; 4 Department of Environmental and Occupational Health Medical College, National Cheng Kung University, Tainan, Taiwan; 5 Department of Emergency Medicine, Taipei Medical University, Taipei, Taiwan; Indian Institute of Science, India

## Abstract

**Objective:**

The flood after a typhoon may lead to increase in patients with cellulitis of lower limbs. However, the microbiological features of these cases are rarely reported. We conducted a study of patients with lower extremity cellulitis after a typhoon followed in southern Taiwan to study the risk factors of cellulitis and the bacteriological features of the patients.

**Methods:**

We reviewed all the medical records of cellulitis at emergency departments of two teaching hospitals in southern Taiwan 30 days before and after the landing of Typhoon Morakot and collected data on the demographic and bacteriological characteristics. In addition, we evaluated the relationship between the daily number of patients and the rainfall in the Tainan area.

**Results:**

The number of cellulitis patients increased from 183 to 344 during the 30-day period after the typhoon. The number peaked in the third and fourth days and lasted for 3 weeks. The proportion of patients with water immersion of the affected limb was higher after the typhoon (6% vs. 37%, odds ratio [OR]: 9.0, 95% Confidence interval [CI]: 4.7–17.2). We found cultures from the infected limbs with immersion had more polymicrobial (73% vs. 26%, OR: 7.8, 95% CI: 3.2–19.2) and Gram-negative bacilli infection (86% vs. 34%, OR: 11.8, 95% CI: 4.1–34.5).

**Conclusions:**

Flood arose from Typhoon Morakot caused increases in cellulitis patients, which lasted for 3 weeks. Antibiotic treatment that were effective to both Gram-positive cocci and Gram-negative bacilli are recommended for patients with limbs emerged in the water**.**

## Introduction

Floods are uncommon but may high impacts that overwhelm physical infrastructure, human resilience, and social organization. According to the statistical data from the Centre for Research on the Epidemiology of Disasters, flood has been the most frequent natural weather disaster (30.7%) and tended to intensify and increase in occurrence as a consequence of climate change and global warming. [1] The experience from the catastrophic flood caused by hurricane Katrina revealed that skin infections, in addition to acute respiratory illness and gastroenteritis, was among the most common diagnoses in initial noninjury hospital visits. [2] Several studies have correlated excess rainfall directly with waterborne disease outbreaks, mainly gastrointestinal and respiratory diseases. [3], [4] The correlation between flood and lower-extremity cellulitis, however, is rarely reported.

The development of cellulitis can be simplified as three steps–bacterial adherence to host cells, invasion of tissue with evasion of host defenses, and elaboration of toxins. [5] Most areas of skin are dry under normal condition, creating an unfavorable environment for bacterial replication. [6] Floods resulting from excess rainfalls made people prone to immersing lower extremities in water and thereby impaired the cutaneous antimicrobial defense mechanism, which increases the risk of cellulitis regardless of the pre-existing skin conditions or wounds and facilitates the bacteria to penetrate the skin barrier.

A small-scale study in 2007 demonstrated an increase in the number of patients with lower limbs cellulitis following the flood caused by Typhoon Haitung. [7] However, this study only covered the 2-week periods before and after the typhoon and included limited clinical characteristics of only 65 cases. In particular, there were no data on the microbiological features of the patients. Therefore, we conducted a more comprehensive study to evaluate the impact of flood on the incidence of lower extremity cellulitis and the bacteriological characteristics of the patients.

## Methods

### Study Design and Setting

The Tainan area, consisting of the Tainan City and the Tainan County, is located in southwestern Taiwan and has a total population around 1,850,000. We conducted a study of patients who visited the emergency department s (EDs) of two teaching hospitals 30 days before and after the Typhoon Morakot landed on Taiwan in 2009. One of the hospitals is situated next to Tainan City and provides medical service mainly to urban citizens, and the other hospital is in the northern part of Tainan area and serves mostly rural inhabitants. The two hospitals take care of the vast majority of the ED patients in the area, with an average of approximately 17,000 ED visits each month.

Typhoon Morakot caused a record-breaking rainfall of 2748.0 millimeters in the southern Taiwan in the next 3 days following its landing on August 7, 2009. The highest single day regional record was broken on August 8, with a mark up to 1403 millimeters**,** resulting in severe damages to the southern Taiwan with mudslides and floods. [8] Except few inhabitants living in the coastal areas, most immersed inhabitants contacted with fresh water during the flood.

This study was reviewed and approved by the institutional review board of Human Research, Chi-Mei Medical Center. The research assistants collected all the required data from the review of medical records; hided any identifiable details of the patients; and recorded these blind data in a privacy enhanced database. Then the researches were allowed to read and analyze the data. The institutional review board of Human Research, Chi-Mei Medical Center permitted the review process and the obtainment of patients’ written informed consents was waived.

### Patient Selection and Data Collection

We reviewed medical records of all ED visits from July 9 to September 6, 2009, including 30 days before and after the landing of the typhoon. Patients with cellulitis were identified according to the definitions of the National Healthcare Safety Network of the Centers for Disease Control and Prevention. [9] Necrotizing fasciitis, erysipelas, and chronic ulcer with soft tissue infection were also included in the study. A patient with multiple ED visits for the same lesion during the study period was included as one case, so that overrepresentation of certain patients in the data can be avoided.

On each patient, we collected the following Information: demographic characteristics; presence or absence of water immersion of the affected limbs before the onset (W+ or W–); underlying medical illness that could compromised the immune system (including diabetes mellitus, liver cirrhosis, chronic renal disease, malignancy, autoimmune disease, alcoholism, long-term steroid or immunosuppressive therapy, human immunodeficiency virus infection); bacteriological results; medical or surgical treatment; and clinical outcomes regarding the requirements for ward or intensive care unit (ICU) admission and mortality. We also collected data on the results of wound cultures, which were obtained using swabs or needle aspirations from purulent material of infective lesions without preparatory cleansing.

**Figure 1:**
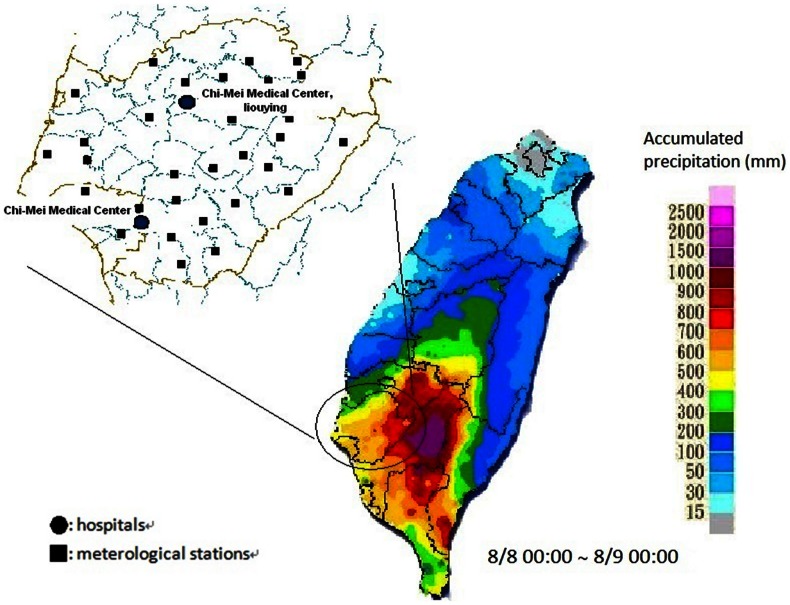
The distribution of meterological stations and hospitals in the Tainan area. •: hospitals ▪: meterological stations.

The amount of daily rainfall at each meteorological station in the Tainan area during the study period was obtained from the southern region weather center. We summed up the amounts and correlate the data with the number of lower extremities cellulitis. The distribution of meteorological stations and the two hospitals was depicted in Figure 1.

### Statistical Analysis

We used Chi-square test and student t-test to evaluate the differences in the clinical characteristics and outcome between the patients before and after the attack of Typhoon Morakot as well as the infected pathogens and categorical variables between the W+ patients and W– patients. For each variable, we calculated the odds ratio (OR) and corresponding 95% confidence interval (CI). The differences in the numbers of patients before and after the typhoon were also evaluated using Chi-square test and the results were presented as with estimated rate ratio (RR) and 95% CI.

## Results

### Incidence of Lower Extremity Cellulitis and Daily Precipitation

**Figure 2:**
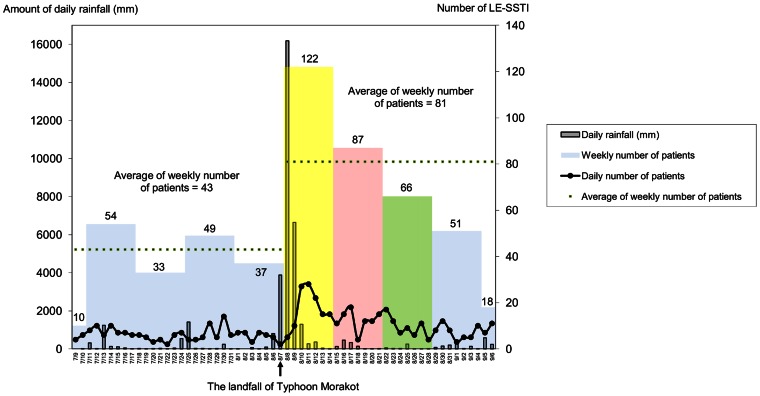
The daily numbers of lower extremity cellulitis patients and the corresponding amount of daily rainfall.

During the study period, we identified a total of 527 qualified patients, including 183 patients before and 344 patients after the typhoon. The number of patients increased after the attack of typhoon at both hospitals (from 109 to 203 and 74 to 141). As T shown in Figure 2, the number of patients peaked on the third and fourth days after the typhoon and remained higher for 3 weeks. The weekly average number of patients during the 4-week period before typhoon was 43 patients. After the typhoon, the numbers of patients in the following 4 weeks were 122, 87, 66, and 51. Using the average weekly case number before typhoon (43) as the baseline, we found that the number of patients increased in the first 3 weeks following the typhoon (RR: 2.8, 95% CI: 2.0–4.0; RR: 2.0, 95% CI: 1.4–2.9; and RR: 1.53, 95% CI: 1.0–2.3, respectively) and returned to the baseline in the fourth week (RR: 1.2, 95% CI: 0.8–1.8).

The total number of all ED visits of the two hospitals were 15790 visits before and 18541 visits after the typhoon. All ED visits raised 17% after flood. Meanwhile, the case of lower extremity cellulitis increased 88% and the increment of lower extremity cellulitis constituted 6% of all increasing ED visits.

### Characteristics of the Patients

Comparing the patients before and after the typhoon, we found no differences in age, gender, underlying medical illness, chronic wound, positive wound culture rate, surgical treatment, and clinical outcomes such as mortality and the requirement for ward or intensive care unit (ICU) admission. (Table 1) The only difference was that the patients after typhoon had higher prevalence of having the affected limbs immersed in water, with an OR of 9.0 (95% CI: 4.7–17.2). Specifically, before the typhoon, the average number of W+ patients was 3 per week, but after the typhoon, the numbers were 61, 36, 17, and 11. Using the average weekly number of W+ patients before typhoon (3) as the baseline, we found the prevalence of W+ patients increased in all four weeks after the typhoon (RR: 20.3, 95% CI: 6.4–64.8; RR: 12.0, 95% CI: 3.7–39.0; RR: 5.7, 95% CI: 1.7–19.3; and RR: 3.7, 95% CI: 1.0–13.1, respectively). As shown in Table 2, there were no differences in clinical characteristics and outcome between the W+ and W– patients.

**Table 1:** The clinical characteristics and outcome between the patients before typhoon and the patients after typhoon.

Characteristics	Before Typhoon (n = 183) no. (%)	After Typhoon (n = 344) no. (%)	OR*	(95% CI†)
Mean Age	51	54	No significant difference**	
Women	74 (40)	130 (38)	0.9	(0.6–1.3)
Underlying medical illness‡	78 (43)	139 (40)	0.9	(0.6–1.3)
Chronic wound	26 (14)	34 (10)	0.7	(0.4–1.1)
Positive wound culture	53 (80)	90 (84)	1.4	(0.6–3.1)
Surgical treatment	30 (16)	38 (11)	0.6	(0.4–1.1)
Water immersion of affected limbs	11 (6)	124 (37)	9.0	(4.7–17.2)
Clinical outcome				
Admission	121 (66)	216 (63)	0.9	(0.6–1.3)
ICU§ requirement	5 (3)	10 (3)	1.1	(0.4–3.2)
Mortality	3 (2)	6 (2)	0.9	(0.2–3.8)

* Odds ratio.

† Confidence interval.

‡ Underlying medical illness: including diabetes mellitus, liver cirrhosis, chronic renal disease, malignancy, autoimmune disease, alcoholism, long-term steroid or immunosuppressive therapy, human immunodeficiency virus infection.

§ Intensive care unit.

** Analyzed by student’s t = test.

**Table 2:** The clinical characteristics and outcome between the W+ and the W– groups*.

Characteristics	W– (n = 390) no. (%)	W+ (n = 137) no. (%)	OR†	(95% CI‡)
Mean Age	51	55	No significant difference**	
Women	151 (39)	53 (39)	1.0	(0.7–1.5)
Underlying medical illness§	167 (43)	50 (36)	0.8	(0.5–1.1)
Surgical treatment	55 (14)	13 (9)	0.6	(0.3–1.2)
Clinical outcome				
Admission	256 (66)	81 (59)	0.8	(0.5–1.1)
ICU¶ requirement	8 (2)	7 (5)	2.6	(0.9–7.2)
Mortality	6 (2)	2 (1)	0.9	(0.2–4.8)

* Presence or absence of water immersion of the affected limbs before the onset.

† Odds ratio.

‡ Confidence interval.

§ Underlying medical illness: including diabetes mellitus, liver cirrhosis, chronic renal disease, malignancy, autoimmune disease, alcoholism, long-term steroid or immunosuppressive therapy, human immunodeficiency virus infection.

¶ Iintensive care unit.

** Analyzed by student’s t = test.

### Bacteriological Analysis

Of the 527 patients, 174 (33%) had a wound or pus specimen submitted for culture (none with both wound and pus specimens), and organisms grew in 143 (82%) specimens. From the 143 patients, 255 bacterial isolates were identified. We excluded 29 patients with chronic wounds (54 isolates) because of their unique bacteriological characters and include the remaining 114 patients (201 isolates ) in the analysis. [10], [11] We found polymicrobial growths in 73% of W+ patients, but in only 26% of W– patients, corresponding to an OR of 7.8 (95% CI: 3.2–11.9). (Table 3) The W+ group had less Gram-positive cocci (GPC) infection (43% vs. 80%; OR: 0.2, 95% CI: 0.1–0.5). Among the GPC, *Staphylococcus* species (spp.), *Streptococcus* spp., and *Enterococcus* spp. were the 3 most frequently isolated organisms. The W+ group had a lower rate of *Staphylococcus aureus* infection (16% vs. 51%; OR: 0.2, 95% CI: 0.1–0.5) but a higher rate of *Enterococcus* spp. Infection (16% vs. 3%; OR: 6.9, 95% CI: 1.3–37.0). In addition, the W+ group had more Gram-negative bacilli (GNB) infection then the W– group (86% vs. 34%, OR: 11.8, 95% CI: 4.1–34.5). The major GNB were *Aeromonas hydrophilia* and *Enterobacteriaceae* (including *Escherichia coli*, *Klebsiella pneumoniae*, *Enterobacter cloacae*, and *Proteus mirabilis*) (Table 3).

**Table 3:** The comparison of isolated bacteria from wound cultures between the W– and the W+ groups*.

	W– (n = 74) no. patient (%)	W+ (n = 37) no. patient (%)	OR†	(95% CI‡)
**Polymicrobial infection** §	19 (26)	27 (73)	7.8	(3.2–19.2)
**Gram-positive cocci**	59 (80)	16 (43)	0.2	(0.1–0.5)
*Staphylococcus aureus*	38(51)	6(16)	0.2	(0.1–0.5)
*Streptococcus* Spp.	16(22)	5(14)	0.6	(0.2–1.7)
*Enterococcus spp.*	2(3)	6(16)	6.9	(1.3–37.0)
*Coaglulase-negative Staphylococcus spp.*	5(7)	2(5)	0.8	(0.1–4.3)
**Gram-negative bacilli**	26 (34)	32 (86)	11.8	(4.1–34.5)
*Aeromonas hydrophila*	3(4)	11(30)	10.0	(2.6–38.5)
*Klebsiella pneumonia*	4(5)	16(43)	13.3	(4.0–43.5)
*Escherichia coli*	2(3)	12(32)	17.2	(3.6–83.3)
*Enterobacter cloacae*	3(4)	8(22)	6.5	(1.6–26.3)
*Proteus mirabilis*	1(1)	6(16)	14.1	(1.6–122.3)
*Pseudomonas aeruginosa*	4(5)	2(5)	1	(0.2–5.7)
*Acinetobacter Baumannia*	7(9)	1(3)	0.3	(0.0–2.2)
*Morexella morganii*	4(5)	3(8)	1.5	(0.3–7.3)
**Others** ¶	5 (7)	1 (3)	0.4	(0.0–3.4)

* Absence or presence of water immersion of the affected limbs before the onset.

† Odds ratio.

‡ Confidence interval.

§ Polymicrobial infection: more than two isolates were identified from wound culture.

¶ Others: including anaerobic bacteria and fungi.

## Discussion

Several studies have found that excess rainfall increased the risk of waterborne diseases, by facilitating entry of human sewage and animal wastes into water systems and transporting of bacteria, viruses, or small parasites. [4], [12] However, most previous studies on waterborne diseases following floods were focused on gastrointestinal and respiratory diseases, and the association between flood and cellulitis was seldom reported. Furthermore, the lack of pre-flood baseline demographic and bacteriological data impeded the assessment of the effects of flood on the occurrence of cellulitis. [13] Our study showed that after the landfall of Typhoon Morakot, the number of lower extremity cellulites patients increased on the next day, peaked around the third and fourth days, and remained elevated in the following 3 weeks. Additionally, though the total ED visits increased after flood, the increment of patients with lower extremity cellulitis is out of proportion to the increment of all ED visits (88% vs. 17%). Whereas the increment of lower extremity cellulitis constituted 6% of all increasing ED visits in our study, some studies observed much higher figures, up to 16% after the Hurricane Katrina for example. [12] Therefore, the local medical service should watch out for the possible outbreak of cellulites after the flood and prepare to deal with the situation for at least three weeks.

Comparing the clinical characteristics of patients before and after typhoon, we found the only difference was that patients observed after typhoon had a higher prevalence of immersing the affected limbs in the water, which appeared to be a major causative factor accounting for the increase of cases after the flood. In addition, the observation that W+ and the W– patients had similar prevalence of having immune compromising diseases implies that water immersion increased the risk of cellulites regardless underlying diseases. Therefore, healthy individuals should still avoid immersing their lower extremities in the flood water.

We found that the W+ group had a higher incidence of polymicrobial infection, the majorities of which were caused by GNB. Among these GNB, *Aeromonas hydrophilia* and *Enterobacteriaceae* (including *Escherichia coli*, *Klebsiella pneumoniae*, *Enterobacter cloacae*, and *Proteus mirabilis*), accounted for 76% of the isolates. This finding was in line with a study of Tsunami survivors [14), which reported 95.5% of GNB with similar isolated pathogens and 71.8% of patients were polymicrobial infection.

GPC, especially *Staphylococcus* spp and *Streptococcus* spp, were considered the most common pathogens of lower extremity cellulitis. In our study, 80% of the W– patients were infected by GPC and the most common pathogens were *Staphylococcus* spp and *Streptococcus* spp. The finding fits the general concept that GPC are the major pathogens of lower extremity cellulitis. On the contrary, only 43% of the W+ patients had GPC infection, and the proportion of *Enterococcus* spp infection increased. A study of metropolitan water supply found that unsterile fresh water may be contaminated by *aeromonas spp* and enteric bacteria, [15] and a review of the literature showed that *Enterococcus* spp, a common enteric bacterium, may be related to cellulites associated with polluted water. [10] These evidence showed that flood may contaminated fresh water and changes the distributions of infected agents for lower extremity cellulitis. Physicians should choose empirical antibiotics that are effective to both GPC and GNB infections, especially *Aeromonas hydrophilia*, and *Enterobacteriaceae* initially and then adjust therapy regiment according to the results of cultures or the patients’ responses to treatment.

The current study has some inherent limitations that are common to retrospective studies. The medical histories were based on the medical records, and therefore, it is possible that some members of the W– groups had immersed their lower extremities into the water. However, the number of such cases should be small because a previous study in Taiwan had reported the association between lower extremity cellulites and flood water, and most physicians in the participating hospitals should be aware of this risk factor. As some of the patients were not the Tainan area, a number of the patients might be from regions not affected by the flood. Nonetheless, because both participating hospitals are within the flooded area and the flood hindered the transportation, the number of such patients should be extremely small, and the effects on our conclusions should be very limited. As in general practice, wound cultures were not performed in all patients, and the incubated organisms from the wound cultures might not be the real pathogens of the cellulitis. Nevertheless, wound culture is still a common and widely accepted method for physicians to identify possible pathogens of cellulitis.

In conclusion, we observed an increase in the occurrence of lower extremity cellulitis after the flood caused by Typhoon Morakot. The peak appeared in the third and fourth days after the flood and remained elevated for 3 weeks. Immersion of lower limbs in floodwater appeared to be a risk factor of lower extremity cellulitis but had similar effects on individuals with and without immune compromising diseases. Because the low extremity cellulitis patients with history of water immersion had high rates of polymicrobial and GNB infections, physicians should choose antibiotics that are effective to both GPC and GNB infections, especially *Aeromonas hydrophilia*, and *Enterobacteriaceae* before further information on the pathogens becomes available.
