# Correction: Epidemiological Characteristics of Lower Extremity Cellulitis after a Typhoon Flood

**DOI:** 10.1371/journal.pone.0171401

**Published:** 2017-01-30

**Authors:** Pei-Chen Lin, Hung-Jung Lin, How-Ran Guo, Kuo-Tai Chen

There is an error in affiliation 5 for author Kuo-Tai Chen. Affiliation 5 should be: Department of Emergency Medicine, School of Medicine, College of Medicine, Taipei Medical University, Taipei, Taiwan.
